# Baseline Levels of Influenza-Specific B Cells and T Cell Responses Modulate Human Immune Responses to Swine Variant Influenza A/H3N2 Vaccine

**DOI:** 10.3390/vaccines8010126

**Published:** 2020-03-13

**Authors:** Lilin Lai, Nadine Rouphael, Yongxian Xu, Amy C. Sherman, Srilatha Edupuganti, Evan J. Anderson, Pamela Lankford-Turner, Dongli Wang, Wendy Keitel, Monica M. McNeal, Kaitlyn Cross, Heather Hill, Abbie R. Bellamy, Mark J. Mulligan

**Affiliations:** 1 The Hope Clinic of the Emory Vaccine Center, Division of Infectious Diseases, Department of Medicine, Emory University School of Medicine, 500 Irvin Court, Decatur, GA 30030, USA; Lilin.Lai@nyulangone.org (L.L.); y.xu@emory.edu (Y.X.); amy.sherman@emory.edu (A.C.S.); sedupug@emory.edu (S.E.); pturn01@emory.edu (P.L.-T.); dwang35@emory.edu (D.W.); 2 Emory Children Center, Departments of Pediatrics and Medicine, Emory University School of Medicine, 2015 Uppergate Drive, Atlanta, GA 30322, USA; evanderson@emory.edu; 3 Departments of Molecular Virology & Microbiology and Medicine, Baylor College of Medicine, One Baylor Plaza, Houston, TX 77030, USA; wkeitel@bcm.edu; 4 Department of Pediatrics, University of Cincinnati College of Medicine, Division of Infectious Diseases, Cincinnati Children’s Hospital Medical Center, Cincinnati, OH 45229, USA; monica.mcneal@cchmc.org; 5 Emmes, 401 N. Washington St., Suite 700, Rockville, MD 20850, USA; kcross@emmes.com (K.C.); hhill@emmes.com (H.H.); abbie.r.bellamy@gmail.com (A.R.B.); 6 New York University Langone Medical Center, Alexandria Center for Life Sciences (West Tower), 430 E 29th St, Room 304, New York, NY 10016, USA; Mark.Mulligan@nyulangone.org

**Keywords:** H3N2v, influenza, vaccine, antibody secreting cells, T cell responses

## Abstract

The cellular immune responses elicited by an investigational vaccine against an emergent variant of influenza (H3N2v) are not fully understood. Twenty-five subjects, enrolled in an investigational influenza A/H3N2v vaccine study, who received two doses of vaccine 21 days apart, were included in a sub-study of cellular immune responses. H3N2v-specific plasmablasts were determined by ELISpot 8 days after each vaccine dose and H3N2v specific CD4+ T cells were quantified by intracellular cytokine and CD154 (CD40 ligand) staining before vaccination, 8 and 21 days after each vaccine dose. Results: 95% (19/20) and 96% (24/25) subjects had pre-existing H3N2v specific memory B, and T cell responses, respectively. Plasmablast responses at Day 8 after the first vaccine administration were detected against contemporary H3N2 strains and correlated with hemagglutination inhibition HAI (IgG: *p* = 0.018; IgA: *p* < 0.001) and Neut (IgG: *p* = 0.038; IgA: *p* = 0.021) titers and with memory B cell frequency at baseline (IgA: r = 0.76, *p* < 0.001; IgG: r = 0.74, *p* = 0.0001). The CD4+ T cells at Days 8 and 21 expanded after prime vaccination and this expansion correlated strongly with early post-vaccination HAI and Neut titers (*p* ≤ 0.002). In an adult population, the rapid serological response observed after initial H3N2v vaccination correlates with post-vaccination plasmablasts and CD4+ T cell responses.

## 1. Introduction

Since 2011, a swine variant of influenza A/H3N2 (H3N2v) has caused 430 infections associated with exposure to pigs at agricultural fairs in 18 different US states with 26 hospitalizations and one death between 2011 and 2018 [1]. The first documented human infection, due to swine H3N2 in North America, occurred in 2005 in an Ontario farm worker [2], with human cases observed mostly from 2011–2013 and a smaller outbreak 2016–2017 [1,3]. H3N2v has had limited human-to-human transmission [4]. It is thought that these H3N2v viruses are derived from a previously circulating human H3N2 virus that spread through the US swine population [2,5]. One-third of 18–49-year-old adults have pre-existing antibodies to the H3N2v, but these are rarely observed in young children in whom most H3N2v infections occurred [4].

Since seasonal influenza vaccines do not provide serological protection against H3N2v, a monovalent subvirion inactivated influenza H3N2v vaccine was developed and tested in 211 adult subjects (Clinicaltrials.gov Registration: NCT01746082) [6]. The vaccine regimen was well-tolerated. Forty percent of subjects had a hemagglutination inhibition (HAI) titer ≥ 40 (seroprotection) before vaccination. Responses were observed at 8 days after the first dose and by 21 days 87% of subjects 18–64 years old achieved an HAI titer ≥ 40. Nearly half of vaccinated adults seroconverted (4-fold rise in antibody titer) and higher geometric mean titers (GMTs) occurred in younger subjects. The second dose did not significantly improve serological responses. In addition, memory B cell (MBC) responses were detected at baseline prior to vaccination, increased after vaccination and were detected against other H3N2 strains. Preexisting H3N2v-specific MBCs positively correlated with antibody responses early after vaccination.

The clinical trial described above provided the opportunity to study the association between pre-existing T cell responses and changes in both T and B cell responses after vaccination in relationship to H3N2v antibody responses. Twenty-five subjects enrolled in the sub-study underwent more detailed cellular immunology testing.

## 2. Materials and Methods 

### 2.1. Study Population

Males and non-pregnant females between the ages of 18 to 64 years were enrolled if they met the eligibility criteria. Subjects with occupational exposure to or direct physical contact with pigs in the past year were excluded. Both the main study and the sub-study protocols were approved by the Emory University Institutional Review Board (IRB00061214 approved 6 December 2012), and written informed consent was provided by subjects prior to study participation. Subjects enrolled at Emory University in the main study were offered optional participation in the immunogenicity sub-study. Subjects were first enrolled in January 2013 and follow-up was completed in October 2013 with subjects receiving 2 doses of influenza A/Minnesota/11/2010 H3N2v subunit vaccine (manufactured by Sanofi Pasteur) administered 21 days apart. 

### 2.2. Viruses and Antigens

The H3N2v vaccine (monovalent influenza A/Minnesota/11/2010) was provided by the Biomedical Advanced Research and Development Authority (BARDA). Antigens used for the HAI assay (A/Minnesota/11/2010 X-203, BPL-Inactivated) and the virus used for the neutralization assay (A/Minnesota/11/2010[H3N2v]) were obtained from the Centers for Diseases Control and Prevention International Reagent Resource (IRR [7]). The recombinant hemagglutinin (rHA) from A/Perth/16/2009 H3N2 was provided by the Biodefense and Emerging Infections Research Repository (NR-19442 [8]). The rHA from A/Victoria/361/2011 H3N2 was provided by IRR (#FR-1059). We selected the A/Perth/16/2009 H3N2 and A/Victoria/361/2011 H3N2 viruses as A/Perth/16/2009 H3N2 was included in inactivated influenza vaccines (IIVs) for the 2010–2011 and 2011–2012 seasons and A/Victoria/361/2011 H3N2 was included in IIVs for the 2012–2013 season. The H3N2v vaccine strain as well as rHA from contemporary strains were used for the antibody secreting cells (ASC) and MBC ELISpot assays, while the H3N2v vaccine strain was used for the intracellular cytokine (ICS) and CD154 staining assay. The amount of rHA or H3N2v used for in vitro stimulation of PBMCs was 200 µg/well.

### 2.3. Peripheral Blood Mononuclear Cell Isolation

Peripheral blood mononuclear cells (PBMCs) were separated from heparinized whole blood using a Ficoll-Hypaque gradient and stored in a liquid nitrogen tank. Cryopreserved PBMCs were thawed in a 37 °C water bath and washed. Before use, cells were counted and checked for viability by Trypan blue dye exclusion.

### 2.4. Serum Hemagglutination Inhibition (HAI) and Neutralization (Neut) Antibody Assays 

HAI and Neut assays were performed at Cincinnati Children’s Hospital Medical Center using previously described methods [9]. 

### 2.5. Antibody-Secreting Cells (Plasmablasts) Assay 

A direct enzyme-linked immunospot (ELISpot) assay was used to enumerate the numbers of total IgG, IgA and IgM ASCs and the H3N2-specific IgG, IgA, and IgM ASCs present in fresh PBMCs, performed as described previously [10]. 96-well ELISpot filter plates (Millipore, Burlington, MA, USA), #MAHA N4510) were coated overnight with purified A/Minnesota/11/2010 H3N2v virus or rHAs of contemporary H3N2 strains in PBS, or goat anti-human Ig (Jackson ImmunoResearch Laboratory, Inc., West Grove, PA, USA). Plates were washed and blocked 2 h at 37 °C prior to use with RPMI 1640 containing 10% fetal calf serum, 100 units/mL of penicillin G, and 100 μg/mL of streptomycin (Gibco), referred to as complete medium. Freshly isolated PBMC were suspended in complete medium distributed into ELISpot plates and incubated overnight at 37 °C in a CO_2_ incubator. ASCs were detected with biotinylated anti-human IgG, IgM, or IgA antibody (Jackson ImmunoResearch Laboratory Inc., West Grove, PA, USA) followed by incubation with Avidin-D-HRP conjugate (Vector Laboratories, Burlingame, CA, USA), and then developed using AEC substrate (3 amino-9 ethyl-carbazole; Sigma-Aldrich, St. Louis, MO, USA). Developed plates were scanned and analyzed using an automated ELISpot counter (Cellular Technologies, Ltd., Shaker Heights, OH, USA). 

### 2.6. H3N2 Specific CD4+ T Cells by Intracellular Cytokine and CD154 Staining Assay

ICS and CD154 staining assay were performed, as previously described [11]. Cryopreserved PBMCs, from vaccinated subjects, were thawed, stimulated for 18 h with A/Minnesota/11/2010 H3N2v subunit vaccine and αCD28/CD49d costimuli (all 1 μg/mL) in the presence of Protein Transport Inhibitor Cocktail (eBioscience, San Diego, CA, USA). After stimulation, PBMCs were stained with Live Dead dye (Biolegend San Diego, CA, USA) before intracellular staining with antibodies against CD3 (SP34-2), CD4 (L200), IL-2 (MQ1-17H12), and CD154 (TRAP1) from BD Biosciences and IFN-gamma (4S.B3) and IL-21 (3A3-N2.1) from eBioscience. PBMCs were then analyzed with an LSR Fortessa instrument (BD Biosciences, Franklin Lakes, NJ, USA). The responses to medium alone were subtracted from antigen-stimulated values at each time point. The frequency of total H3N2-specific CD4+ T cells was calculated by summing the frequency of CD4+ T cells producing all non-overlapping permutations of the cytokines and CD154 tested (e.g., CD154, IFN-gamma, IL-2 and IL-21) using Boolean and logical gates with FlowJo (v9.7.6). 

### 2.7. Memory B Cell (MBC) Assay

MBCs were also tested by ELISpot after polyclonal stimulation of PBMC in vitro as previously described [10] by incubating cryopreserved PBMCs at 5 × 10^6^ cells per mL in R-10 supplemented with 1/100,000 pokeweed mitogen extract (PWM), 6 μg/mL phosphothiolated CpG ODN-200626 (Integrated DNA technologies, Coralville, IA, USA), and 1/10,000 fixed Staphylococcus aureus Cowan (SAC) (Sigma, St. Louis, MO, USA) for 6 days. Eight replicate wells were cultured per subject. Stimulated cells were harvested, washed and applied to ELISpot filter plates which had been coated overnight with A/Minnesota/11/2010 H3N2v subunit vaccine or rHAs of contemporary H3N2 strains in PBS, or goat anti-human Ig (Jackson ImmunoResearch Laboratory Inc., West Grove, PA, USA). Total and virus-specific IgG-secreting MBCs were quantified by ELISpot assay as described above in plasmablasts ELISpot assay. Insufficient PBMCs did not allow quantification of MBCs in 5 out of 25 subjects.

### 2.8. Statistical Methods

As an exploratory sub-study, the sample size was limited to the number of subjects enrolled in the parent clinical trial at the Emory site and not designed to test a statistical hypothesis. Analyses includes descriptive and graphical summaries and Pearson correlations. Confidence intervals for binomial proportions were calculated using the exact Clopper-Pearson method.

Post-vaccination responses were compared with baseline using Wilcoxon signed-rank test. HAI, Neut, IgA, IgM and IgG responses were log-transformed for analysis. Statistical significance was considered at a level alpha = 0.05, without adjustment for multiple comparisons. Analyses were performed using SAS 9.3 or higher (SAS Institute Inc., Cary, NC, USA). 

## 3. Results

### 3.1. Demographics

Twenty-five subjects were enrolled in this immunogenicity sub-study. Baseline characteristics of subjects included in this subset are summarized in Table 1. Subjects had a mean age of 38 years (range, 22–63), approximately half (44%) were female, and 48% were white and 32% were Black/African American. Most subjects (76%) had received trivalent IIV in one or both of the prior seasons (2011–2012 and/or 2012–2013). These seasonal vaccines contained either the A/Perth/16/2009 (H3N2)-like virus (2011–2012 season) or the A/Victoria/361/2011(H3N2)-like virus (2012–2013 season). All subjects had met criteria for the per protocol population analyses in the main study.

### 3.2. Preexisting Cross-Reactive Antibody and Expansion of the Antibody Response

**Figure 1:**
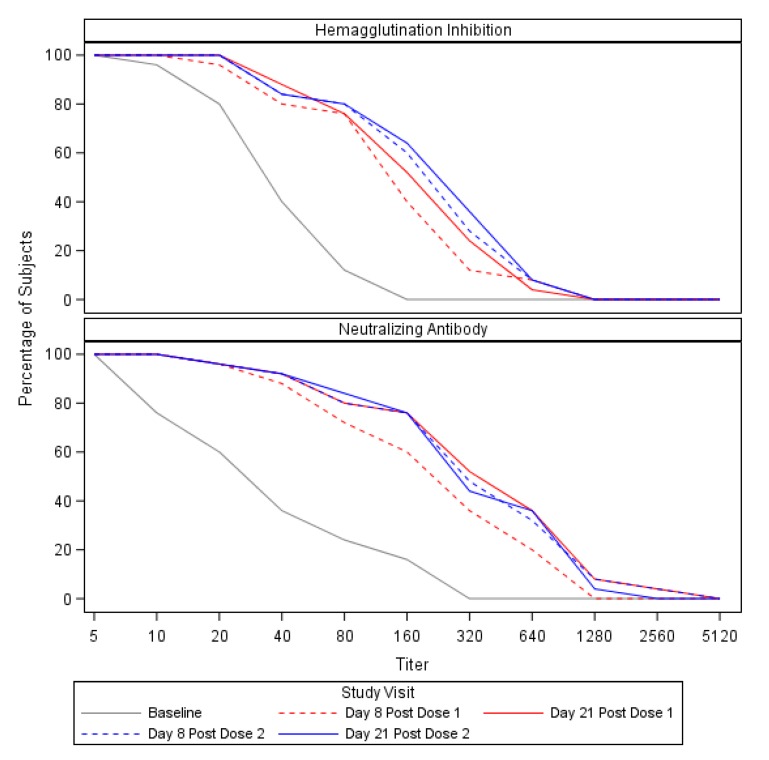
Reverse cumulative distribution curves of serum hemagglutination inhibition and neutralizing antibodies following immunization with H3N2v vaccine.

HAI and Neut antibody responses against H3N2v for the 25 subjects included in the sub-study (Figure 1 and Table 2) were consistent with those observed in the main study for those between the ages of 18–64 years [6]. Subjects had modest antibody titers at baseline (Day 0 HAI GMT = 28 and Neut GMT = 29). On Day 8 (after the first vaccination), GMTs of HAI and Neut antibodies increased from baseline levels to 92 and 178, respectively. By Day 21 after the first vaccination, these GMTs had further increased to 118 and 286 for HAI and Neuts, respectively. HAIs and Neuts did not appreciably change after the booster dose; seroconversion occurred in about half of subjects by Day 8, and did not change significantly at Day 21 and following the second dose of H3N2v vaccine (Table 2, Supplementary Tables S1 and S2) or by demographics and baseline characteristics of participants (Supplementary Table S3). 

### 3.3. Plasmablast Responses Correlated with HAI and Neut Titers and Were Detected against Seasonal H3N2 Antigens

At baseline no subjects had detectable variant or seasonal H3N2-specific IgG or IgA ASCs (plasmablasts) in blood as expected. At Day 8, the number of influenza-specific IgG-secreting plasmablasts against the H3N2v/Minnesota/2010 strain had a geometric mean (GM) of 205 per million PBMCs (95% CI: 104–402; range 0–9477). At Day 8, the number of influenza-specific IgA-secreting plasmablasts against the H3N2v/Minnesota/2010 strain had a GM of 56 per million PBMCs (95% CI: 31–102; range 0–509) (Table 3). Plasmablasts against seasonal H3N2 strains were also detected at Day 8. There were fewer influenza-specific IgA-secreting plasmablasts against the seasonal Victoria/2011 antigen (GM 21; range: 0–99 per million PBMC) and the seasonal Perth/2009 antigen (GM 24; range: 0–126 per million PBMC) than observed against the H3N2v antigen. There were also fewer influenza-specific IgG secreting plasmablasts for the seasonal Victoria/2011 (GM 84; range: 0 to 2187 per million PBMC) and Perth/2009 (GM 58; range: 0–1377 per million PBMC) strains. No subject had detectable H3N2v-specific or seasonal H3N2-specific IgA or IgG plasmablasts in blood at Day 8 following the second dose of H3N2v vaccine, consistent with the observation that the second dose did not significantly enhance antibody responses. 

Total IgM secreting ASCs were detected, as well as the total IgA and IgG (data not shown), however, H3N2 specific IgM ASCs were not detected, as their peak likely occurred prior to the detection of H3N2 specific IgG and IgA ASCs. The magnitudes of H3N2v-specific IgG- and IgA-secreting ASCs at Day 8 correlated with H3N2v HAI and Neut antibody titers at Day 8 (Table 4). 

**Figure 2:**
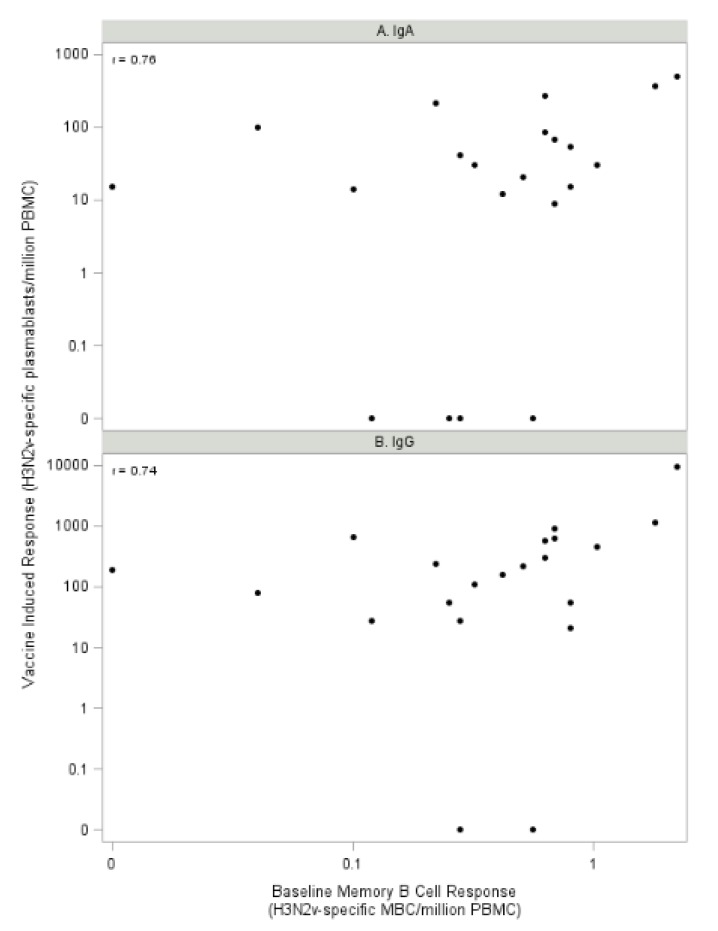
Percentage of MBCs producing H3N2v-specific IgG at baseline (Day 0) correlates with Day 8 H3N2v-specific plasmablast response (**A**: IgA and **B**: IgG).

In addition, the baseline frequency of IgG-secreting MBCs that produced antibody against H3N2v correlated with the Day 8 frequency of plasmablasts (IgA: r = 0.76, *p* < 0.001; IgG: r = 0.74, *p* = 0.0001) (Figure 2A,B), which is consistent with the finding that pre-existing H3N2v-specific MBCs positively correlated with early increases in vaccine-induced Ab we reported previously (6).

### 3.4. Expansion of Circulating Antiviral CD4+ T Cells against H3N2v Correlated with Antibody Responses

**Figure 3:**
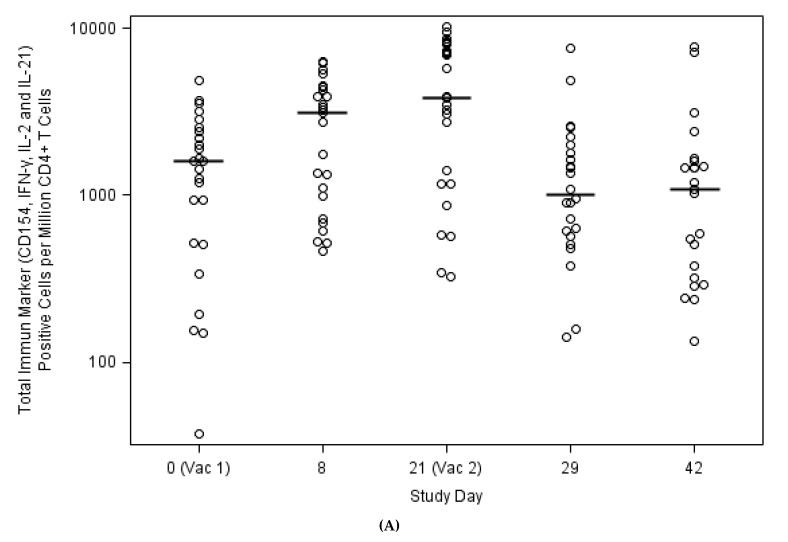

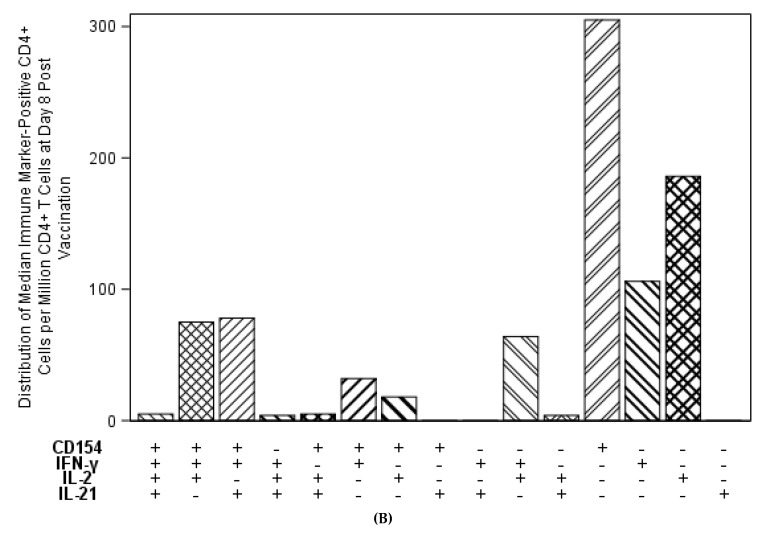
CD4+ T cell responses induced by H3N2v vaccination. (**A**) Temporal H3N2v CD4+ T cells among vaccinees as determined by 4 immune marker secretion (CD154, IFN-γ, IL-2 and IL-21), and (**B**) Immune marker profile of CD4+ T cells at Day 8.

Four immune markers (CD154, IFN-γ, IL-2 and IL-21) were used to identify H3N2v specific CD4+ T cells. At baseline, immune marker positive CD4+ T cells were detected in 24 out of 25 subjects (median 1584; range: 37–4815 CD4+ T cells specific for H3N2v per 10^6^ CD4+ T cells) (Figure 3A). After the first dose of vaccination, the magnitude of CD4+ T cells responses increased at Day 8 (median: 3084; range: 462–6337) and at Day 21 (median: 3783; range: 323–10,193 per 10^6^ CD4+ T cells). Both Day 8 and 21 H3N2v-specific responses were significantly higher compared to baseline values (*p* < 0.001 at both Days 8 and 21), demonstrating that vaccine-specific CD4+ T-cell responses occurred following H3N2v vaccination. Interestingly, the magnitude of H3N2v-specific CD4+ T cells declined to levels similar to pre-vaccination following the second dose [median 1010; range 140–7549 at Day 8 post second dose (*p* = 0.41) and median 1079; range: 133–7694 at Day 21 post second dose, (*p* = 0.34)] (Figure 3A), suggesting that the T cell response was not further boosted by the second vaccine dose, similar to the lack of significant boost in the humoral response. 

It appeared that a lower baseline memory H3N2-specific CD4+ T cell response likely resulted in a higher HAI or Neut post-vaccination titer at all time points checked. This inverse correlation did not reach statistical significance, possibly due to the relatively small number of subjects in the sub-study (Table 5). However, the change from baseline in H3N2v-specific CD4+ T cells (e.g., vaccine induced CD4+ T cell response) correlated with all post-vaccination Neut antibody titers and early HAI titers (Days 8, 21 and 29) (Table 6).

In addition, the CD4+ T cell response is polyfunctional. as determined by the frequency of CD4+ T cells producing 1, 2, 3 or 4 immune markers among CD154, IFN-gamma, IL-2 and IL-21 (Figure 3B).

## 4. Discussion

Influenza A viruses have been isolated from swine since the 1930s. H1N1 virus was the predominant subtype, isolated from U.S. swine until the late 1990s, when human H3N2 viruses spread widely among North American pigs. Since then, triple re-assortant swine influenza A/H3N2 viruses emerged and began circulating in pigs containing genes from human, swine and avian influenza viruses [5]. Transmission of H3N2v strains to humans resulted in 18 outbreaks in the US [1] with more than 400 cases. However, sustained human-to-human transmission has not occurred. The A/H3N2 antigens contained in recent seasonal influenza vaccines do not provide serological protection in young children against this H3N2 variant strain [12,13,14], hence the need to develop a vaccine specifically targeting H3N2v. The current study offers a new understanding of the cellular responses to a monovalent unadjuvanted vaccine against an emergent influenza strain (H3N2v). 

Similar to the primary study [6], our study demonstrates that the administration of a single standard dose of monovalent IIV induces an early and robust antibody response against H3N2v. This recall response could be explained by the fact that H3N2v is similar to H3N2 strains that circulated in the 1990s. In our adult population (all born prior to 1995), subjects appear to have been primed with antigenically related strains as 40% of subjects had an HAI titer ≥40 at baseline against H3N2v, similar to findings in other serosurveillance studies [12,13,15]. Moreover, in this cohort, circulating H3N2v MBCs and memory T cells were both detected at baseline in the vast majority of subjects. 

It is known that pre-vaccination serological status has a large impact on the immune response to seasonal influenza vaccination and seroconversion rates are negatively associated with past influenza vaccinations, baseline HAI Ab titers ≥ 40 and baseline HAI Ab titers [16,17]. Here, we focused on evaluating the association between B and T cell responses and antibody responses to H3N2v. 

We previously reported that pre-existing H3N2v MBCs correlated with the Day 8 fold-change of HAI antibody [6], and further assessment of the cellular response now shows the positive correlation between MBCs and the frequency of plasmablasts. The antibody titers detected after vaccination correlated with the ASCs at Day 8, as previously described [18]. In addition, the kinetics of the ASCs in our study were similar to those observed after seasonal IIV, where the detection of circulating influenza virus-specific ASCs is transient, peaking around one week (7–12 days) after immunization and then rapidly decreasing, so that very few if any influenza virus-specific ASCs are detected one month after immunization [19,20]. Influenza virus-specific IgG and IgA ASCs are typically detected after influenza. While the majority of adults and older children have demonstrable IgG ASC responses, the IgA ASC responses were detected in less than one-third to half of subjects [21]. This was not the case in our study, as the majority of subjects had both IgG (88%) and IgA (80%) ASC responses at Day 8 after the first vaccination.

Though some evidence suggests that preexisting influenza-specific CD4+ T cells correlate with disease protection in an influenza human challenge model [22], other studies have shown that baseline CD4+ T cell responses negatively correlate with antibody response after influenza vaccine [23,24]. A better understanding of the role of CD4+ T cell responses is needed for novel influenza vaccines. In the current study, CD4+ T cells specific to H3N2v antigens were present before vaccination and expanded shortly after vaccination. These findings indicate that both naive and memory CD4+ T cells were rapidly recruited in response to a single dose of novel H3N2v vaccine. We found a trend of negative correlation between the magnitude of baseline CD4+ T cell responses and all subsequent HAI and Neut responses. In our study, the expansion of the vaccine-induced CD4+ T cell responses also correlated with increases in HAI and Neut antibody titers similar to data from pandemic H1N1 vaccine studies [23,24]. The change from baseline in H3N2v-specific CD4+ T cells at Day 8 and D21 correlated strongly with early post-vaccination antibody responses. 

Increased in ASCs and MBCs detection to contemporary H3N2 strains such as A/Perth/16/2009 H3N2 and A/Victoria/361/2011 H3N2 after H3N2v vaccination [25]. In pediatric studies [26], the B cell responses to antigenically drifted H3N2 viruses during a vaccine mismatch season showed evidence of cellular cross-reactivity without observing a cross reactive serological neutralizing antibody responses. 

The study had several limitations. A major limitation of our study is that we did not conduct HAI and Neut antibody assays against the contemporary strains. We had a limited number of subjects resulting in limited power to detect correlations. We included only subjects between the ages of 18–64 years [6]. Therefore, we have no data on the cellular immune responses in elderly and children. Since children under nine years of age require two doses of vaccine to demonstrate an adequate serological response, a better understanding of cellular immunity to influenza vaccines is needed in the pediatric population [14].

## 5. Conclusions

Our data demonstrated pre-existing memory B and T cells could modulate antibody response to vaccination of newly emerged variant influenza strain, and may have implications for the design of vaccination strategies against potential future influenza pandemics and the identification of early markers of vaccine efficacy. Since there is a great interest in defining an early biomarker that will predict a successful response to a vaccine, the presence of antigen-specific MBCs at baseline [6], the magnitude of Day 8 changes in antigen-specific CD4+ T cells, and Day 8 plasmablasts responses after prime vaccination were all identified as potential markers of serological responses to H3N2v vaccination.

## Figures and Tables

**Table 1:** Demographic and baseline characteristics of participants.

Characteristics	Values (Sample Size *n* = 25)
Age	
Mean (SD)	38.4 (14.4)
Median [Min, Max]	30.0 [22.0, 63.0]
Gender—*n* (%)	
Female	11 (44)
Male	14 (56)
Race—*n* (%)	
Asian	4 (16)
Black/African American	8 (32)
Multi-Racial	1 (4)
White	12 (48)
Prior IIV ^1^ Receipt—*n* (%)	
None	5 (20)
2012/13 IIV only	1 (4)
2011/12 IIV only	7 (28)
Both 2011/12 + 2012/13 IIV	11 (44)
Unknown prior IIV	1 (4)

^1^ IIV: Inactivated Influenza Vaccine.

**Table 2:** Serum Hemagglutination Inhibition and Neutralizing antibody levels and percent of subjects seroconverting after the first and second doses of H3N2v vaccine.

Antibody Titers	Measured Metric	Day 0 (Pre-Vaccination)	Day 8 (Post Vaccination 1)	Day 21 (Post Vaccination 1, Immediately Pre-Vaccination 2)	Day 29 (Day 8 Post Vaccination 2)	Day 42 (Day 21 Post Vaccination 2)
Hemagglutination Inhibition (HAI)	Geometric Mean Titer (95% CI)	27.5 (19.8, 38.3)	91.9 (59.1, 142.8)	117.9 (79.8, 174.2)	126.4 (81.7, 195.6)	135.5 (86.2, 213.0)
	Seroconversion ^1^—*n* (%)	NA	13 (52.0)	14 (56.0)	13 (52.0)	13 (52.0)
Neutralizing Antibody (Neut)	Geometric Mean Titer (95% CI)	28.5 (16.0, 50.7)	178.0 (103.6, 306.0)	285.5 (162.2, 502.5)	276.9 (157.8, 486.1)	258.3 (149.7, 445.6)
	Seroconversion ^1^—*n* (%)	NA	12 (48.0)	13 (52.0)	13 (52.0)	13 (52.0)

^1^ Seroconversion was defined as HAI or Neut 4-fold increase from baseline to a titer ≥ 40.

**Table 3:** Influenza strain-specific plasmablasts ^1^ per million PBMCs at Day 8 post first vaccination.

Measured Metric	IgG			IgA		
Strains	H3N2v Minnesota/2010	H3N2 Perth/2009	H3N2 Victoria/2011	H3N2v Minnesota/2010	H3N2 Perth/2009	H3N2 Victoria/2011
Median (min, max)	162 (0, 9477)	9.0 (0, 1377)	9.0 (0, 2187)	30.0 (0, 509)	0.0 (0, 126)	0.0 (0, 99)
Geometric Mean (95% CI)	204.7 (104.2, 402.2)	58.0 (20.4,164.7)	84.0 (32.1, 219.9)	56.1 (30.8, 101.8)	23.7 (12.9, 43.4)	21.2 (9.1, 49.3)
Number of Subjects with a Positive Response (%)	22 (88)	14 (56)	13 (52)	20 (80)	9 (36)	7 (28)

**Table 4:** Correlation between magnitude of Day 8 H3N2v-specific plasmablasts and Day 8 serum antibody responses after the first vaccination.

Plasmablasts	Hemagglutination Inhibition Antibody (*p*-Value)	Neutralizing Antibody (*p*-Value)
IgG	0.469 (0.018)	0.417 (0.038)
IgA	0.647 (<0.001)	0.457 (0.021)

**Table 5:** Correlation of baseline CD4+ T cell response with post vaccination antibody responses.

Measured Metrics	Hemagglutination Inhibition Titer (*p*-Value)				Neutralizing Antibody Titer (*p*-Value)			
Days	Day 8	Day 21	Day 29	Day 42	Day 8	Day 21	Day 29	Day 42
CD154, IFN-γ, IL-2 or IL-21 expression CD4+ T-Cells on Day 0	−0.293 (0.155)	−0.307 (0.135)	−0.290 (0.159)	−0.207 (0.321)	0.169 (0.421)	−0.216 (0.300)	−0.239 (0.249)	−0.267 (0.197)

**Table 6:** Correlation of post vaccination CD4+ T cell responses with serum antibody responses.

Measured Metrics	Hemagglutination Inhibition Titer (*p*-Value)				Neutralizing Antibody Titer (*p*-Value)			
CD154, IFN-γ, IL-2 or IL-21 expression CD4+ T Cells change from baseline (Day 0)	**Day 8**	**Day 21**	**Day 29**	**Day 42**	**Day 8**	**Day 21**	**Day 29**	**Day 42**
Day 8–Day 0	0.582 (0.002)	0.649 (<0.001)	0.526 (0.007)	0.265 (0.200)	0.621 (<0.001)	0.859 (<0.001)	0.870 (<0.001)	0.602 (<0.001)
Day 21–Day 0		0.667 (<0.001)	0.542 (0.005)	0.295 (0.153)		0.865 (<0.001)	0.874 (<0.001)	0.618 (<0.001)
Day 29–Day 0			0.525 (0.008)	0.279 (0.187)			0.909 (<0.001)	0.687 (<0.001)
Day 42–Day 0				0.280 (0.196)				0.679 (<0.001)

## Supplementary Materials

The following are available online at https://www.mdpi.com/2076-393X/8/1/126/s1, Table S1: Summary of HAI antibody titer and seroconversion, Table S2: Summary of neutralization antibody titer and seroconversion, Table S3: HAI and MN response by demographic and baseline characteristics of participants.

## References

[1] Case Count: Detected U.S. Human Infections with H3N2v by State since August 2011|Swine/Variant Influenza (Flu). https://www.cdc.gov/flu/swineflu/h3n2v-case-count.htm.

[2] Olsen C.W., Karasin A.I., Carman S., Li Y., Bastien N., Ojkic D., Alves D., Charbonneau G., Henning B.M., Low D.E. (2006). Triple Reassortant H3N2 Influenza A Viruses, Canada, 2005. Emerg. Infect. Dis..

[3] Blanton L., Wentworth D.E., Alabi N., Azziz-Baumgartner E., Barnes J., Brammer L., Burns E., Davis C.T., Dugan V.G., Fry A.M. (2017). Update: Influenza Activity—United States and Worldwide, May 21–September 23, 2017. MMWR Morb. Mortal. Wkly. Rep..

[4] Epperson S., Jhung M., Richards S., Quinlisk P., Ball L., Moll M., Boulton R., Haddy L., Biggerstaff M., Brammer L. (2013). Human Infections with Influenza A(H3N2) Variant Virus in the United States, 2011–2012. Clin. Infect. Dis..

[5] Zhou N.N., Senne D.A., Landgraf J.S., Swenson S.L., Erickson G., Rossow K., Liu L., Yoon K., Krauss S., Webster R.G. (1999). Genetic Reassortment of Avian, Swine, and Human Influenza A Viruses in American Pigs. J. Virol..

[6] Keitel W.A., Jackson L.A., Edupuganti S., Winokur P.L., Mulligan M.J., Thornburg N.J., Patel S.M., Rouphael N.G., Lai L., Bangaru S. (2015). Safety and Immunogenicity of a Subvirion Monovalent Unadjuvanted Inactivated Influenza A(H3N2) Variant Vaccine in Healthy Persons ≥ 18 Years Old. J. Infect. Dis..

[7] International Reagent Resource. https://www.internationalreagentresource.org/.

[8] BEI Resources. http://www.beiresources.org.

[9] Rowe T., Abernathy R.A., Hu-Primmer J., Thompson W.W., Lu X., Lim W., Fukuda K., Cox N.J., Katz J.M. (1999). Detection of Antibody to Avian Influenza A (H5N1) Virus in Human Serum by Using a Combination of Serologic Assays. J. Clin. Microbiol..

[10] Crotty S., Aubert R.D., Glidewell J., Ahmed R. (2004). Tracking human antigen-specific memory B cells: A sensitive and generalized ELISPOT system. J. Immunol. Methods.

[11] Spensieri F., Borgogni E., Zedda L., Bardelli M., Buricchi F., Volpini G., Fragapane E., Tavarini S., Finco O., Rappuoli R. (2013). Human Circulating influenza-CD4^+^ ICOS1^+^IL-21^+^ T Cells Expand after Vaccination, Exert Helper Function, and Predict Antibody Responses. Proc. Natl. Acad. Sci. USA.

[12] Houser K.V., Katz J.M., Tumpey T.M. (2013). Seasonal Trivalent Inactivated Influenza Vaccine Does Not Protect against Newly Emerging Variants of Influenza A (H3N2v) Virus in Ferrets. J. Virol..

[13] Waalen K., Kilander A., Dudman S.G., Ramos-Ocao R., Hungnes O. (2012). Age-dependent prevalence of antibodies cross-reactive to the influenza A(H3N2) variant virus in sera collected in Norway in 2011. Eurosurveillance.

[14] Munoz F.M., Anderson E.J., Bernstein D.I., Harrison C.J., Pahud B., Anderson E., Creech C.B., Berry A.A., Kotloff K.L., Walter E.B. (2019). Safety and immunogenicity of unadjuvanted subvirion monovalent inactivated influenza H3N2 variant (H3N2v) vaccine in children and adolescents. Vaccine.

[15] Skowronski D.M., Janjua N.Z., De Serres G., Purych D., Gilca V., Scheifele D.W., Dionne M., Sabaiduc S., Gardy J.L., Li G. (2012). Cross-reactive and Vaccine-Induced Antibody to an Emerging Swine-Origin Variant of Influenza A Virus Subtype H3N2 (H3N2v). J. Infect. Dis..

[16] Sasaki S., He X.-S., Holmes T.H., Dekker C.L., Kemble G.W., Arvin A.M., Greenberg H.B. (2008). Influence of Prior Influenza Vaccination on Antibody and B-Cell Responses. PLoS ONE.

[17] Sacadura-Leite E., Sousa-Uva A., Rebelo-de-Andrade H. (2012). Antibody response to the influenza vaccine in healthcare workers. Vaccine.

[18] Halliley J.L., Kyu S., Kobie J.J., Walsh E.E., Falsey A.R., Randall T.D., Treanor J., Feng C., Sanz I., Lee F.E.-H. (2010). Peak frequencies of circulating human influenza-specific antibody secreting cells correlate with serum antibody response after immunization. Vaccine.

[19] Cox R.J., Brokstad K.A., Zuckerman M.A., Wood J.M., Haaheim L.R., Oxford J.S. (1994). An early humoral immune response in peripheral blood following parenteral inactivated influenza vaccination. Vaccine.

[20] El-Madhun A.S., Cox R.J., Søreide A., Olofsson J., Haaheim L.R. (1998). Systemic and Mucosal Immune Responses in Young Children and Adults after Parenteral Influenza Vaccination. J. Infect. Dis..

[21] Sasaki S., Jaimes M.C., Holmes T.H., Dekker C.L., Mahmood K., Kemble G.W., Arvin A.M., Greenberg H.B. (2007). Comparison of the Influenza Virus-Specific Effector and Memory B-Cell Responses to Immunization of Children and Adults with Live Attenuated or Inactivated Influenza Virus Vaccines. J. Virol..

[22] Wilkinson T.M., Li C.K.F., Chui C.S.C., Huang A.K.Y., Perkins M., Liebner J.C., Lambkin-Williams R., Gilbert A., Oxford J., Nicholas B. (2012). Preexisting influenza-specific CD4+ T cells correlate with disease protection against influenza challenge in humans. Nat. Med..

[23] He X.-S., Holmes T.H., Sasaki S., Jaimes M.C., Kemble G.W., Dekker C.L., Arvin A.M., Greenberg H.B. (2008). Baseline Levels of Influenza-Specific CD4 Memory T-Cells Affect T-Cell Responses to Influenza Vaccines. PLoS ONE.

[24] Nayak J.L., Fitzgerald T.F., Richards K.A., Yang H., Treanor J.J., Sant A.J. (2013). CD4^+^ T-Cell Expansion Predicts Neutralizing Antibody Responses to Monovalent, Inactivated 2009 Pandemic Influenza A(H1N1) Virus Subtype H1N1 Vaccine. J. Infect. Dis..

[25] Bangaru S., Nieusma T., Kose N., Thornburg N.J., Finn J.A., Kaplan B.S., King H.G., Singh V., Lampley R.M., Sapparapu G. (2016). Recognition of influenza H3N2 variant virus by human neutralizing antibodies. JCI Insight.

[26] Kim J.H., Mishina M., Chung J.R., Cole K.S., Nowalk M.P., Martin J.M., Spencer S., Flannery B., Zimmerman R.K., Sambhara S. (2016). Cell-Mediated Immunity Against Antigenically Drifted Influenza A(H3N2) Viruses in Children During a Vaccine Mismatch Season. J. Infect. Dis..

