# Supplementary material for: Baseline Levels of Influenza-Specific B Cells and T Cell Responses Modulate Human Immune Responses to Swine Variant Influenza A/H3N2 Vaccine
Source: Vaccines (Basel). 2020 Mar 13;8(1):126. doi: 10.3390/vaccines8010126 (PMC7157591; doi:10.3390/vaccines8010126)
Supplement: Supplementary file 1 [file vaccines-08-00126-s001.pdf]

vaccines

MDPI

Article

Baseline Levels of Influenza-Specific B Cells and T Cell Responses Modulate Human Immune Responses to Swine Variant Influenza A/H3N2 Vaccine

Table S1. Summary of HAI antibody titer and seroconversion.

Subject	Day 0 (Vac 1)	Day 8			Day 21 (Vac 2)			Day 28			Day 42		
	Titer	Titer	Ratio to Baseline	SC[1]	Titer	Ratio to Baseline	SC[1]	Titer	Ratio to Baseline	SC[1]	Titer	Ratio to Baseline	SC[1]
ID01	113.14	80	0.7	N	80	0.7	N	160	1.4	N	160	1.4	N
ID02	56.57	80	1.4	N	160	2.8	N	160	2.8	N	160	2.8	N
ID03	10	20	2	N	28.28	2.8	N	20	2	N	20	2	N
ID04	40	80	2	N	56.57	1.4	N	160	4	Y	80	2	N
ID05	40	160	4	Y	160	4	Y	160	4	Y	160	4	Y
ID06	20	80	4	Y	160	8	Y	160	8	Y	320	16	Y
ID07	28.28	28.28	1	N	40	1.4	N	20	0.7	N	20	0.7	N
ID08	10	226.27	22.6	Y	320	32	Y	320	32	Y	160	16	Y
ID09	20	80	4	Y	452.55	22.6	Y	640	32	Y	640	32	Y
ID10	10	20	2	N	20	2	N	20	2	N	20	2	N
ID11	20	640	32	Y	320	16	Y	452.55	22.6	Y	452.55	22.6	Y
ID12	113.14	452.55	4	Y	160	1.4	N	160	1.4	N	320	2.8	N
ID13	5	10	2	N	20	4	N	20	4	N	20	4	N
ID14	40	80	2	N	80	2	N	80	2	N	80	2	N
ID15	10	80	8	Y	113.14	11.3	Y	80	8	Y	160	16	Y
ID16	56.57	80	1.4	N	80	1.4	N	80	1.4	N	80	1.4	N
ID17	20	160	8	Y	320	16	Y	320	16	Y	320	16	Y
ID18	113.14	226.27	2	N	226.27	2	N	320	2.8	N	320	2.8	N
ID19	40	160	4	Y	160	4	Y	226.27	5.7	Y	320	8	Y
ID20	20	20	1	N	40	2	N	40	2	N	40	2	N
ID21	28.28	640	22.6	Y	640	22.6	Y	640	22.6	Y	452.55	16	Y
ID22	28.28	160	5.7	Y	160	5.7	Y	160	5.7	Y	160	5.7	Y
ID23	20	80	4	Y	80	4	Y	80	4	Y	80	4	Y
ID24	40	160	4	Y	320	8	Y	320	8	Y	640	16	Y
ID25	28.28	40	1.4	N	113.14	4	Y	113.14	4	Y	160	5.7	Y

[1] SC = Seroconversion

Table S2. Summary of neutralization antibody titer and seroconversion.

Subject	Day 0 (Vac 1)	Day 8			Day 21 (Vac 2)			Day 28			Day 42		
	Titer	Titer	Ratio to Baseline	SC[1]	Titer	Ratio to Baseline	SC[1]	Titer	Ratio to Baseline	SC[1]	Titer	Ratio to Baseline	SC[1]
ID01	254	317	1.2	N	334	1.3	N	304	1.2	N	405	1.6	N
ID02	165	210	1.3	N	412	2.5	N	501	3	N	644	3.9	N
ID03	20	54	2.7	N	54	2.7	N	47	2.4	N	45	2.3	N
ID04	148	175	1.2	N	159	1.1	N	309	2.1	N	154	1	N
ID05	5	337	67.4	Y	632	126.4	Y	548	109.6	Y	372	74.4	Y
ID06	5	267	53.4	Y	708	141.6	Y	677	135.4	Y	748	149.6	Y
ID07	5	21	4.2	N	35	7	N	32	6.4	N	20	4	N
ID08	10	735	73.5	Y	655	65.5	Y	594	59.4	Y	319	31.9	Y
ID09	5	79	15.8	Y	696	139.2	Y	671	134.2	Y	670	134	Y
ID10	27	39	1.4	N	75	2.8	N	75	2.8	N	82	3	N
ID11	26	699	26.9	Y	685	26.3	Y	801	30.8	Y	658	25.3	Y
ID12	5	5	1	N	5	1	N	5	1	N	5	1	N
ID13	11	42	3.8	N	232	21.1	Y	179	16.3	Y	234	21.3	Y
ID14	79	135	1.7	N	208	2.6	N	214	2.7	N	281	3.6	N
ID15	14	156	11.1	Y	284	20.3	Y	156	11.1	Y	314	22.4	Y
ID16	102	278	2.7	N	188	1.8	N	238	2.3	N	161	1.6	N
ID17	67	655	9.8	Y	1797	26.8	Y	1551	23.1	Y	957	14.3	Y
ID18	315	567	1.8	N	659	2.1	N	653	2.1	N	658	2.1	N
ID19	242	654	2.7	N	686	2.8	N	659	2.7	N	680	2.8	N
ID20	37	51	1.4	N	67	1.8	N	61	1.6	N	74	2	N
ID21	5	1227	245.4	Y	3693	738.6	Y	3448	689.6	Y	1559	311.8	Y
ID22	13	565	43.5	Y	541	41.6	Y	440	33.8	Y	292	22.5	Y
ID23	33	192	5.8	Y	229	6.9	Y	232	7	Y	213	6.5	Y
ID24	75	433	5.8	Y	823	11	Y	705	9.4	Y	1206	16.1	Y
ID25	22	111	5	Y	215	9.8	Y	254	11.5	Y	281	12.8	Y

[1] SC = Seroconversion

Table S3. HAI and MN response by demographic and baseline characteristics of participants.

Characteristics		HAI Response at Day 42		MN Response at Day 42	
	N	GMT (95% CI)	Seroconversion - % (95% CI)	GMT (95% CI)	Seroconversion - % (95% CI)
Age					
18 – 49 (N=X)	19	121.70 (72.93-203.09)	47.4 (24.4-71.1)	268.22 (157.44-456.93)	42.1 (20.3-66.5)
50+ (N=X)	6	190.27 (52.06-695.48)	66.7 (22.3-95.7)	229.21 (28.39-1850.35)	83.3 (35.9-99.6)
Gender					
Female	11	128.33 (60.45-272.45)	63.6 (30.8-89.1)	285.47 (140.25-581.05)	63.6 (30.8-89.1)
Male	14	141.37 (74.15-269.55)	42.9 (17.7-71.1)	238.76 (98.58-578.30)	42.9 (17.7-71.1)
Race					
Asian	4	160.00 (44.77-571.78)	50.0 (6.8-93.2)	414.29 (154.26-1112.65)	50.0 (6.8-93.2)
Black	8	174.48 (67.37-451.86)	50.0 (15.7-84.3)	331.89 (113.31-972.14)	37.5 (8.5-75.5)
Multi-Racial	1	160.00 (NC)	100.0 (2.5-100.0)	281.00 (NC)	100.0 (2.5-100.0)
White	12	106.79 (49.00-232.72)	50.0 (21.1-78.9)	185.38 (69.20-496.61)	58.3 (27.7-84.8)
Prior IIV[1] Receipt					
None	5	171.48 (35.91-818.89)	60.0 (14.7-94.7)	437.74 (109.60-1748.34)	40.0 (5.3-85.3)
2011/12 or 2012/13 IIV	20	127.73 (77.04-211.75)	50.0 (27.2-72.8)	226.37 (119.73-428.00)	55.0 (31.5-76.9)

[1] IIV: Inactivated Influenza Vaccine; NC = Not calculated due to insufficient data

© 2020 by the authors. Licensee MDPI, Basel, Switzerland. This article is an open access article distributed under the terms and conditions of the Creative Commons Attribution (CC BY) license (http://creativecommons.org/licenses/by/4.0/).
